# A Pedagogical Note: Use of Telepractice to Link Student Clinicians to Diverse Populations

**DOI:** 10.5195/ijt.2016.6190

**Published:** 2016-07-01

**Authors:** STACY GALLESE CASSEL, AMY J. HADLEY EDD

**Affiliations:** DEPARTMENT OF COMMUNICATION DISORDERS, STOCKTON UNIVERSITY, GALLOWAY, NEW JERSEY, USA

**Keywords:** Cultural competence, Speech-language pathology, Telepractice

## Abstract

Telepractice is the application of telecommunications technology to the delivery of telehealth services via the online connection of clinicians, clients, and patients for assessment, intervention, or consultation. This article describes a pilot project in which speech-language pathology students in a university training program gained experience in working with culturally diverse preschool students using telepractice technology. The preschool students benefited by making gains in communication skills, while the university students acquired competency in the use of telepractice and in working with children whose cultural and linguistic backgrounds were outside of their experience. To assess the training experience, a Likert-scale survey administered to student clinicians revealed a high degree of satisfaction and improved familiarity with the use of telepractice, and an increased comfort level working with multi-cultural populations.

University students completing certification requirements for the American Speech-Language and Hearing Association (ASHA) as speech-language pathologists must complete 400 clock hours of supervised clinical experience (ASHA, 2014). According to ASHA’s Standards and Implementation Procedures for the Certificate of Clinical Competence in Speech-Language Pathology, “Supervised practicum must include experience with client/patient populations across the life span and from culturally/linguistically diverse backgrounds” (ASHA, 2014, Standard V). Matteliano and Stone (2014) recognized the disparity of primarily Caucasian healthcare providers when compared to an increasingly ethnically diverse patient population, identifying a subsequent need for increased clinician education in cultural competence.

Students need to understand the cultural and socioeconomic factors that impact the daily lives of their clients as well as the beliefs and values of the communities in which the clients live (Matteliano & Stone, 2014).

Steed (2014) identified racial biases toward African-Americans among allied health professional students (including speech-language pathology students) after administering the Racial Argument Scale to students in a southern university. Steed concluded that discussing cultural diversity in the classroom is not enough to overcome bias, but that “students must be actively engaged in immersive partnerships with community health care consumers” (Steed, 2014, p. 85).

A pilot study examining such partnerships was conducted by Barton, Moore and Squires (2012), wherein students received direct experience working with culturally diverse early intervention populations and were deemed to have mastered the targeted cultural competencies. Competency was determined by course grades, student practicum competencies, caregiver satisfaction, parent and child outcomes, and job placement after graduation. The authors concluded that current and future therapists should demonstrate an understanding of and appreciation for the cultures of families they serve (Barton, Moore, & Squires, 2012).

Speech-language pathologists work in a variety of settings. According to the most recently published membership survey by the American Speech-Language Hearing Association (ASHA, 2013), over half of all speech-language pathologists work in school settings. In a separate survey of strictly school-based speech-language pathologists, over 70% of respondents indicated that their caseloads include English language learners (Brook, 2012). When asked to rate how qualified they felt working with multi-cultural populations on a 5-point scale, approximately a third of respondents rated themselves as “not at all qualified” (Brook, 2012, p. 32).

Given the increased diversity of the children that speech-language pathology students will likely encounter when entering the workforce, it is crucial that during their clinical training, they gain direct clinical experience with multi-cultural individuals whose backgrounds differ from their own. For some university clinics, providing experiences with culturally and linguistically diverse populations within the same geographic region as the program may pose a challenge (ASHA, 2014). In addition, gaining access to certain types of clinical practicum sites with the appropriate levels of supervision is an ongoing challenge for university programs; this is likely secondary to qualified personnel shortages and budget cuts (Dudding, Grogan-Johnson, & Murphree-Holden, 2010; Mancinelli & Amster, 2015). The use of telepractice may provide a solution by potentially offering students clinical experience with culturally and linguistically diverse clients.

According to the American Speech-Language-Hearing Association, telepractice is “the application of telecommunications technology to the delivery of speech-language pathology and audiology professional services at a distance by linking clinician to client/patient or clinician to clinician for assessment, intervention, or consultation” (ASHA, n.d., para.1). The two most readily accessed modes of telepractice involve synchronous methods (conducted via an interactive audio and video connection in real time to create an experience similar to an in-person setting) and asynchronous methods (involving the transmission of captured data). By applying telecommunications technology to deliver professional services from a distance, “there are no inherent limits to where telepractice can be implemented” (ASHA, n.d., para. 4). Research to date indicates that the use of telepractice yields clinical outcomes and patient satisfaction consistent with results obtained through traditional in-person service delivery models (ASHA, n.d.).

There is a growing base of evidence demonstrating the success of telepractice as a method of speech-language therapy service delivery in the schools (Grogan-Johnson, Alvares, Rowan, & Creaghead, 2010; Scheideman-Miller, Clark, Smeltzer, Carpenter, Hodge, & Prouty, 2002; McCullough, 2001). In a partnership between schools in Ohio and Kent State University, graduate students successfully provided speech and language services to children in a rural school setting (Alvares et al., 2014). By utilizing telepractice, university clinics responsible for training student practitioners have the opportunity to align themselves with schools and other clinical settings to: (a) provide quality speech and language services to underserved populations; (b) provide university students with clinical experiences that were previously more difficult to acquire (e.g., working with multi-cultural populations); and, (c) provide services in settings (e.g., public/private schools) that are experiencing clinician shortages.

## METHODS

### PARTICIPANTS

Participants in the telepractice program included eight undergraduate university speech-language pathology students enrolled in their first clinical experience. Each student was supervised by a licensed and certified speech-language pathologist. Four preschool-aged children received speech and language services twice weekly for eight weeks through a telepractice partnership between the university and their preschool.

### CLINICAL SETTING

Stockton University operates a speech and hearing clinic that serves approximately 35 clients per semester. The clients receive treatment from student clinicians under the direction of licensed and certified (in speech-language pathology) faculty members. The students periodically receive experience in conducting speech, language and hearing screenings at community health fairs and at local preschool and daycare centers located near the university. The clinic is located in a suburban area in southern New Jersey.

During the 2013–2014 academic year, the university program received a request to provide speech, language, and hearing screenings to approximately 50 preschool aged children in a non-profit, early learning center (ELC) located approximately 60 miles from campus. The ELC is located in an inner city in the Mid-Atlantic region and is unique in the fact that its students represent children at risk secondary to abuse or neglect. The ELC is accredited by NAEYC, the National Association for the Education of Young Children. More than 75% of the children attending the ELC are classified as an ethnic minority, either speaking a non-standard dialect of English or residing in a bilingual home. Thus, the children attending the ELC were both ethnically and linguistically diverse, with cultural and socio-economic backgrounds different from those of the university students.

In response to the request for screenings, a faculty member accompanied several students to the ELC to conduct the screenings. At that time, four children were identified as needing full evaluations. Results from these evaluations determined that speech-language therapy was indicated for all four children. Three of the children demonstrated speech sound disorders and the fourth child demonstrated both a speech sound disorder and deficits in oral language. The children were initially referred for services through the local school district, but the ELC director was informed that they would be placed on a waiting list for services due to personnel shortages.

Due to the distance between the ELC and the university, it was not feasible for the children to attend therapy at the university speech and hearing clinic. As a result, a pilot project was developed through which the preschool-aged children could receive free services through the university via telepractice.

### EQUIPMENT

Telepractice equipment was set up both at the clinic and the ELC. Both sites were already equipped with computers and an internet connection. Additionally, each site was equipped with a Logitech HD Pro Webcam C920 (1080p Widescreen) for teleconferencing and recording capabilities. The teleconferencing application chosen was VSee, presumed to support HIPAA compliance. VSee is defined as “a proprietary low-bandwidth, group video chat and screen-sharing software tool being used as a HIPAA compliant alternative” to other videoconferencing platforms (VSee.com, 2015, para.1).

### SERVICE DELIVERY

Eight student clinicians, working in pairs, were assigned to the children. These students collaborated on developing therapy plans and materials under the direction of supervising faculty members. During the therapy sessions, one student would interact with the preschool student via the telecommunication system, while the other would document quantitative data on the child’s performance in therapy. The university students would alternate the task of interacting with the preschool child and collecting data. At the end of the intervention period, supervisors used course grades based on pre-determined clinical competencies and child outcomes as measures of each student’s ability to provide services via telepractice to a multi-cultural pediatric population.

Each week, quantitative treatment plans were developed and were shared electronically (through encrypted, HIPAA-compliant email) with staff at the ELC. Materials such as worksheets or stimulus pictures were also electronically sent to the ELC on a weekly basis. Some of the learning activities involved matching, sound discrimination and identification, imitation, and labeling. The students successfully adapted both therapy materials and behavior management plans typically used in in-person settings for the telepractice mode of service delivery.

At the ELC, an adult facilitator was present during the treatment sessions, seated next to the child. The facilitator’s role included: (a) physical presentation of therapy materials; (b) clarification of instructions between the child and the clinician; and, (c) providing additional skill modeling as needed. Each session was scheduled for twenty minutes, twice weekly. Telecommunication was additionally used to communicate with the children’s teachers. Written progress reports and home practice activities were sent home periodically to families throughout the intervention period.

### STUDENT SURVEY OF CLINICAL TELEPRACTICE SERVICE DELIVERY

The student clinicians completed a 5-point ascending Likert-scale survey that targeted two parameters: (1) student-clinician familiarity and satisfaction with the use of telepractice; and, (2) student-clinician familiarity and satisfaction with working with multi-cultural populations outside of their clinical experience. Survey questions are presented in Appendix A.

## RESULTS

### PARTICIPANT OUTCOMES

Based on quantitative post-evaluation findings and progress notes, all four children demonstrated improvement over the course of the academic year on their speech and language objectives. At the end of the academic year, all of the children successfully transitioned to kindergarten, where any residual speech or language needs could continue to be addressed via telepractice. Detailed participant data are not included since clinical training is the focus of this pedagogical note.

### STUDENT CLINICIAN OUTCOMES

An informal assessment of the participating student clinicians (via an ascending Likert scale) revealed a high degree of satisfaction and improved familiarity with the use of telepractice, and an increased comfort level working with multi-cultural populations. This is demonstrated in the table below, which summarizes assessment findings.

Based on the success of the pilot year, the telepractice partnership with the ELC continued the following year; four additional children were identified as having speech-language deficits and were added to the telepractice caseload. The goal of the clinic supervisors was to add an additional four student clinicians to the telepractice program.

## CHALLENGES AND LIMITATIONS

One challenge encountered was the limited bandwidth speed of the ELC. The video transmission of the session would occasionally freeze, while the audio portion would remain intact. Also, there was some initial turnover in the role of the facilitator. Turnover was reduced by identifying a university student who lived near the ELC and was interested in gaining experience working with children.

A limitation of this investigation was the small sample size of student clinicians involved in the project (four); however, the clinic supervisors wanted to ensure continuity of care by having the same student clinician provide services to the student. As this was the clinic’s first attempt to initiate this pilot program, the attending supervisors also felt that monitoring a small group of students would enable the team to document and troubleshoot initial difficulties more readily, thereby maximizing the quality of service provided.

## DISCUSSION AND CONCLUSIONS

Speech-language pathologists entering the workforce must be prepared to meet the needs of a culturally and linguistically diverse client population. The standards for certification of the American Speech-Language Hearing Association (2014) require that all university students studying speech-language pathology have experience with clients from culturally and linguistically diverse backgrounds. According to the (2014), by 2050, about half of the United States population will identify themselves as members of a racial or ethnic minority group. Yet, members of minority groups in the United States continue to have greater numbers of individuals who reside in poverty, are less educated, and have poorer access to healthcare. States with large ethnically diverse populations, including Florida, Texas, Illinois, New York, and California, are the same states that are most likely to report shortages of qualified speech-language pathologists (Agency for Healthcare Research and Quality, 2014).

Having direct clinical experience is related to students’ self-efficacy as clinicians (Pasupathy & Bogschutz, 2013). Self-efficacy includes an individual’s belief that he or she is able to achieve certain tasks and is related to occupational choice (Pasupathy & Bogschutz, 2013). Providing direct experience with clients whose cultural and linguistic backgrounds differ from those of speech-language pathology students may not only help them to master certification competencies but to gain confidence and proficiency in working with diverse populations.

Telepractice holds much promise as an educational tool for university programs. The cost to employ a telepractice program is relatively small. The advantages include increased exposure to diverse client populations, decrease in time and money spent on travel for both clients and service providers, and access to services by those who may otherwise be underserved.

## Figures and Tables

**Table 1:** Student Clinician Ratings

STUDENT CLINICIAN RATING OF A TELEPRACTICE EXPERIENCE FEATURING A MULTI-CULTURAL PEDIATRIC POPULATION	
**1) Prior to this clinical experience, I was unfamiliar with telepractice.**	

**Clinician**	**Response**

Clinician 1	Strongly agree (5)

Clinician 2	Disagree (2)

Clinician 3	Neutral (3)

Clinician 4	Strongly agree (5)

**2) After this clinical experience, I am more familiar with telepractice.**	

**Clinician**	**Response**

Clinician 1	Strongly agree (5)

Clinician 2	Agree (4)

Clinician 3	Agree (4)

Clinician 4	Agree (4)

**3) The pediatric clients I served in this practicum are from an ethnic/socioeconomic group different from my own**.	

**Clinician**	**Response**

Clinician 1	Agree (4)

Clinician 2	Agree (4)

Clinician 3	Strongly agree (5)

Clinician 4	Strongly agree (5)

**4) Prior to this clinician experience, I felt knowledgeable about working with children from an ethnic/socioeconomic culture different from my own.**	

**Clinician**	**Response**

Clinician 1	Neutral (3)

Clinician 2	Agree (4)

Clinician 3	Agree (4)

Clinician 4	Agree (4)

**5) After this clinical experience, I felt more knowledgeable about working with children from an ethnic/socioeconomic culture different from my own.**	

**Clinician**	**Response**

Clinician 1	Strongly agree (5)

Clinician 2	Agree (4)

Clinician 3	Agree (4)

Clinician 4	Agree (4)

**6) Overall, I feel I learned a lot about a culture different from my own.**	

**Clinician**	**Response**

Clinician 1	Strongly agree (5)

Clinician 2	Agree (4)

Clinician 3	Agree (4)

Clinician 4	Disagree(2)

**7) Overall, I feel that this telepractice clinical experience was a positive experience.**	

**Clinician**	**Response**

Clinician 1	Strongly agree (5)

Clinician 2	Agree (4)

Clinician 3	Agree (4)

Clinician 4	Agree (4)

**8) I would participate in a telepractice clinical experience again.**	

**Clinician**	**Response**

Clinician 1	Strongly agree (5)

Clinician 2	Agree (4)

Clinician 3	Agree (4)

Clinician 4	Agree (4)

Note. 5-Point Scale (5=strongly agree, 4=agree, 3=neutral, 2=disagree, 1=strongly disagree)

## APPENDIX A. Student clinician ratings of telepractice experience featuring a multi-cultural pediatric population

Instructions: Using the 5-point scale below, please rate your clinical experience based on the following statements:

1. Prior to this clinical experience, I was unfamiliar with telepractice.
  5 = Strongly agree
  4 = Agree
  3 = Neutral
  2 = Disagree
  1 = Strongly disagree
  N/A = Not applicable
2. After this clinical experience, I am more familiar with telepractice.
  5 = Strongly agree
  4 = Agree
  3 = Neutral
  2 = Disagree
  1 = Strongly disagree
  N/A = Not applicable
3. The pediatric clients I served in this practicum are from an ethnic/socioeconomic group different from my own.
  5 = Strongly agree
  4 = Agree
  3 = Neutral
  2 = Disagree
  1 = Strongly disagree
  N/A = Not applicable
4. Prior to this clinical experience, I felt knowledgeable about working with children from an ethnic/socioeconomic culture different from my own.
  5 = Strongly agree
  4 = Agree
  3 = Neutral
  2 = Disagree
  1 = Strongly disagree
  N/A = Not applicable
5. After this clinical experience, I feel more knowledgeable about working with children from an ethnic/socioeconomic culture different from my own.
  5 = Strongly agree
  4 = Agree
  3 = Neutral
  2 = Disagree
  1 = Strongly disagree
  N/A = Not applicable
6. Overall, I feel I learned a lot about a culture different from my own
  5 = Strongly agree
  4 = Agree
  3 = Neutral
  2 = Disagree
  1 = Strongly disagree
  N/A = Not applicable
7. Overall, I feel that this telepractice clinical experience was a positive experience.
  5 = Strongly agree
  4 = Agree
  3 = Neutral
  2 = Disagree
  1 = Strongly disagree
  N/A = Not applicable
8. I would participate in a telepractice clinical experience again.
  5 = Strongly agree
  4 = Agree
  3 = Neutral
  2 = Disagree
  1 = Strongly disagree
  N/A = Not applicable

## References

[b1-ijt-pg41] Agency for Healthcare Research and Quality (2014). Disparities in healthcare quality among racial and ethnic minority groups: Selected findings from the 2010 National Healthcare Quality and Disparities Reports.

[b2-ijt-pg41] Alvares R, Grogan-Johnson S, Grime J, Brigle M, Dalrymple J, Houk K (2014). Practical strategies for implementing and managing a school-based telepractice program in a rural school. American Speech-Language Hearing Association Annual Convention.

[b3-ijt-pg41] ASHA Telepractice.

[b4-ijt-pg41] ASHA (2013). Highlights of the 2013 ASHA Membership Survey: SLPs.

[b5-ijt-pg41] ASHA (2014). 2014 Standards and Implementation Procedures for the Certificate of Clinical Competence in Speech-Language Pathology.

[b6-ijt-pg41] Barton EE, Moore HW, Squires JK (2012). Preparing speech language pathology students to work in early childhood. Topics in Early Childhood Special Education.

[b7-ijt-pg41] Brook G (2012). Data at a glance: SLPs changing caseloads. ASHA Leader.

[b8-ijt-pg41] Dudding C, Grogan-Johnson S, Murphree-Holden C (2010). Academic program capacity building. American Speech-Language Hearing Association Annual Convention.

[b9-ijt-pg41] Grogan-Johnson S, Alvares R, Rowan L, Creaghead N (2010). A pilot study comparing the effectiveness of speech language therapy provided by telemedicine with conventional on-site therapy. Journal of Telemedicine and Telecare.

[b10-ijt-pg41] Mancinelli JM, Amster BJ (2015). Rethinking Clinical Education. ASHA Leader.

[b11-ijt-pg41] Matteliano MA, Stone JH (2014). Cultural competence education in university rehabilitation programs. Journal of Cultural Diversity.

[b12-ijt-pg41] McCullough A (2001). Viability and effectiveness of teletherapy for pre-school children with special needs. International Journal of Language & Communication Disorders.

[b13-ijt-pg41] Pasupathy R, Bogschutz RJ (2013). An investigation of graduate speech-language pathology students’ SLP clinical self-efficacy. Contemporary Issues in Communication Science and Disorders.

[b14-ijt-pg41] Scheideman-Miller C, Clark PG, Smeltzer SS, Carpenter J, Hodge B, Prouty D (2002). Two year results of a pilot study delivering speech therapy to students in a rural Oklahoma school via telemedicine.

[b15-ijt-pg41] Steed R (2014). Caucasian allied health students’ attitudes toward African-Americans: Implications for instruction and research. ABNF Journal.

[b16-ijt-pg41] VSee (2015). https://vsee.com/.

